# Enzymatically Functionalized Composite Materials Based on Nanocellulose and Poly(Vinyl Alcohol) Cryogel and Possessing Antimicrobial Activity

**DOI:** 10.3390/ma12213619

**Published:** 2019-11-04

**Authors:** Aysel Aslanli, Nikolay Stepanov, Tatyana Razheva, Elena A. Podorozhko, Ilya Lyagin, Vladimir I. Lozinsky, Elena Efremenko

**Affiliations:** 1 Faculty of Chemistry, Lomonosov Moscow State University, Moscow 119991, Russia; ayselaslanli@mail.ru (A.A.); na.stepanov@gmail.com (N.S.); lyagin@mail.ru (I.L.); 2 N.M.Emanuel Institute of Biochemical Physics RAS, Moscow 119334, Russia; 3 A.N.Nesmeyanov Institute of Organoelement Compounds, Russian Academy of Sciences, Moscow 119334, Russia; razhevatanya@gmail.com (T.R.); epodorozhko@mail.ru (E.A.P.); loz@ineos.ac.ru (V.I.L.)

**Keywords:** immobilized hexahistidine-tagged organophosphorus hydrolase, poly(vinyl alcohol) cryogel, bacterial cellulose, β-lactam antibiotic, antimicrobial peptides, bactericidal activity

## Abstract

In the present work, innovative composite biomaterials possessing bactericidal properties and based on the hexahistidine-tagged organophosphorus hydrolase (His_6_-OPH) entrapped in the poly(vinyl alcohol) cryogel (PVA-CG)/bacterial cellulose (BC) were developed. His_6_-OPH possesses lactonase activity, with a number of N-acyl homoserine lactones being the inducers of Gram-negative bacterial resistance. The enzyme can also be combined with various antimicrobial agents (antibiotics and antimicrobial peptides) to improve the efficiency of their action. In this study, such an effect was shown for composite biomaterials when His_6_-OPH was entrapped in PVA-CG/BC together with β-lactam antibiotic meropenem or antimicrobial peptides temporin A and indolicidin. The residual catalytic activity of immobilized His_6_-OPH was 60% or more in all the composite samples. In addition, the presence of BC filler in the PVA-CG composite resulted in a considerable increase in the mechanical strength and heat endurance of the polymeric carrier compared to the BC-free cryogel matrix. Such enzyme-containing composites could be interesting in the biomedical field to help overcome the problem of antibiotic resistance of pathogenic microorganisms.

## 1. Introduction

It is now well recognized that the problem of antibiotic resistance of bacteria is of great significance [1]. Such resistance can progress by the mechanism of “quorum sensing” (QS), which is the ability of bacterial cells to interact with each other within the same population and to initiate a shift in the biochemical status of cells that leads to resistance. Both Gram-positive and Gram-negative (G(-)) bacteria use different signaling molecules as QS inducers. The most pathogenic G(-) bacteria are known to prefer N-acyl homoserine lactones (AHLs) as such signaling molecules and certain AHLs are typical for the bacterial species [2]. An efficient approach to overcome G(-) bacterial resistance is the decomposition of AHLs via enzymatic action [3]. Therefore, lactonases that directly break down the ester bond in the lactone ring of most AHLs are of special interest [4]. Hexahistidine-tagged organophosphorus hydrolase (His_6_-OPH) has been found to be one such enzyme. It has a wide specificity with a number of AHLs in addition to high catalytic activity toward toxic organophosphorus compounds [5,6]. His_6_-OPH in noncovalent complexes with various antimicrobial agents (antibiotic and, particularly, antimicrobial peptides) has been shown to improve the activity of each other [7,8,9]. As a result, the enzyme has a wider substrate spectrum toward different AHLs in such combinations, and its stability is increased [7,8,9].

From the practical standpoint, thorough immobilization of His_6_-OPH has its own merits [10,11] as the final catalytically active biomaterials can be more convenient for biotechnological and biomedical applications [12,13]. Therefore, an appropriate carrier is also important.

For this purpose, the so-called poly(vinyl alcohol) cryogel (PVA-CG) [14,15] was chosen in this work as the carrier’s matrix for His_6_-OPH immobilization. Such hydrophilic macroporous gels are known to be formed as a result of cryogenic processing of concentrated PVA solutions via their consecutive freezing, exposing in a frozen state, and thawing [16]. Nontoxicity and biocompatibility, in combination with excellent mechanical, diffusion, and thermophysical properties, make PVA-CG attractive matrices for use in medicine, biotechnology, and bioengineering [13,17,18,19]. The physicochemical properties of these cryogels can be controlled by varying the concentration of the initial polymer solution, changing the regime of low-temperature exposure and thawing, and introducing various soluble and insoluble modifiers. In particular, nontoxic cellulose-based disperse fillers improve the gel strength considerably [20] and are of interest for the development of biomedical materials. Among such insoluble modifiers are micro- and nanofibers of bacterial cellulose (BC) [21,22]. For instance, the mechanical properties of PVA-CG/BC composites, which were studied as potential artificial cartilages, turned out to be very similar to the rheological characteristics of native articular cartilages [18,23].

BC is a fibrous biopolymer obtained from the culture broth of various bacterial strains, among which acetic acid bacteria are the most productive microorganisms capable of BC biosynthesis in various nutrient media [21]. In contrast to plant cellulose, BC does not contain hemicellulose and lignin admixtures and therefore does not require separation and purification from them. BC has high water capacity and is a biodegradable, biocompatible, and nontoxic biopolymer with unique physical and mechanical properties, such as high mechanical strength, elasticity, permeability for liquids and gases, etc. The structure and properties of BC depend on cultivation conditions and can be modified by aerating and mixing of the nutrient medium and by changing its composition, pH, temperature, etc. [24]. When BC is incorporated in various polymeric matrices, they impart new set of properties to the composites, thus allowing such innovative materials to be applied in various areas [25,26], including pharmaceutics and biomedicine [22]. It has previously been shown that BC can also be used to immobilize various enzymes [27,28,29,30]. However, studies on the effect of BC production and cultivation conditions on the properties of immobilized enzymes are rather limited [27].

The driving force of the current work was to combine the properties of the above-described three components—namely, the catalytic abilities of His_6_-OPH hydrolyzing AHLs, macroporosity of PVA-CG matrix, and enzyme-adsorbing properties of BC—into new bioactive composite materials. In other words, the aim of the study was to form an immobilized biocatalyst possessing good operational characteristics and exhibiting certain antimicrobial and catalytic properties. Yet another task was to evaluate the bactericidal characteristics of similar composites prepared with additives of β-lactam antibiotic or antimicrobial peptides, both forming noncovalent complexes with His_6_-OPH [7,8,9]. The development of such composite biomaterials with bactericidal properties can be considered an important task in view of the rapid increase in the number of antibiotic-resistant pathogenic microorganisms and a significant decrease in the effectiveness of action of the known and commonly used antibiotics.

## 2. Results

### 2.1. Preparation of Composite Biomaterials

**Figure 1:**
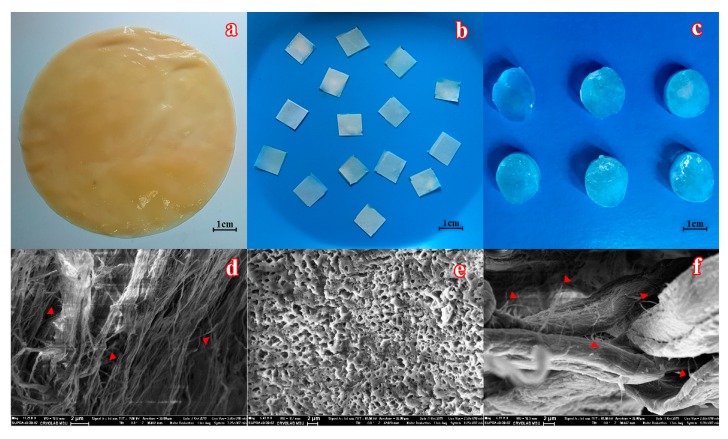
The appearance of a bacterial cellulose (BC) mat biosynthesized by immobilized *K. xylinum* cells in fructose-containing medium (**a**) before and (**b**) after cutting into pieces, which were further used to immobilize the enzyme. (**c**) The general view of poly(vinyl alcohol) cryogel (PVA-CG)/BC composites. SEM images of (**d**) BC, (**e**) PVA-CG, and (**f**) PVA-CG/BC/hexahistidine-tagged organophosphorus hydrolase (His_6_-OPH) composites. Individual nanofibers of BC, marked by red triangles, are seen within the initial material and its composite.

BC samples (Figure 1a) possessing different characteristics were prepared by cultivation of *Komagataeibacter xylinum* B-12429 cells with fructose, glycerol, Jerusalem artichoke hydrolyzate, or beet molasses as the main carbon source (Table 1). BC samples produced with beet molasses or Jerusalem artichoke hydrolyzate had a greater thickness, higher tensile strength, and lower porosity. The carbon sources insignificantly influenced the degree of BC crystallinity. These results are consistent with the data of other researchers [31], who pointed out that BC fibers produced with the hydrolyzate of soybean whey had a 2-fold greater tensile strength compared to BC fibers formed in a standard glucose-containing medium.

Both PVA-CG and PVA-CG/BC composite had rubber-like nonfilled macroporous structures (Figure 1c,f), whose physicomechanical and thermal properties mainly depended on the initial PVA concentration and on the entrapped filler amount in the case of composites (Table 2).

A strengthening effect caused by the BC entrapment in the bulk of PVA-CG was evident. A 1.1 wt % of such filler increased the modulus of elasticity of the material by 4-fold, and the fusion temperature grew by ~12 °C compared to the nonfilled PVA-CG. Such properties of immobilized enzyme carrier are promising due to its possible application in biotechnological processes under real conditions, e.g., in reactors with intense stirring and at elevated temperatures.

The sample prepared using BC in a medium containing fructose had the highest activity (Table 3). Therefore, this BC was further used in the PVA-CG/BC/His_6_-OPH composite with or without the addition of antimicrobial agents.

Similar values (68%−73%) of initial activity were earlier observed after covalent immobilization of OPH on chemically modified plant cellulose microfibers [32]. However, in the case of bacterial cellulose, the chemical modification of which is extremely difficult due to the small pore size and low availability of polymer for modification by chemical agents, the sorption variant of enzyme immobilization is the most appropriate.

### 2.2. Catalytic Activity of Composite Biomaterials

**Figure 2:**
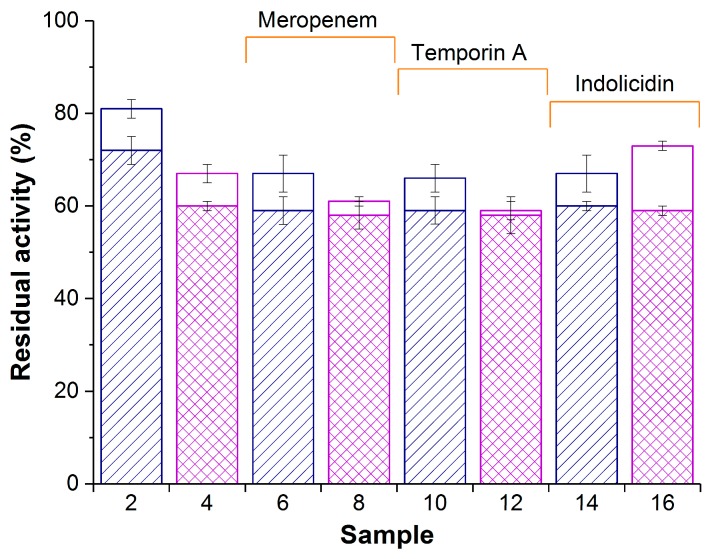
Enzymatic activity of composite biomaterials PVA-CG/His_6_-OPH (samples 2,6,10, and 14) and PVA-CG/BC/His_6_-OPH (samples 4,8,12, and 16) in the absence (samples 2 and 4) or in the presence of meropenem (samples 6 and 8), temporin A (samples 10 and 12), and indolicidin (samples 14 and 16). White and patterned bars represent activity at zero time and after 24 h, respectively. The same samples prepared without His_6_-OPH were used as controls and had no measurable hydrolase activity. Initial activity of free His_6_-OPH was accepted as 100%.

The enzyme activity of PVA-CG/His_6_-OPH and PVA-CG/BC/His_6_-OPH composites with or without different antimicrobial agents (β-lactam antibiotic meropenem or antimicrobial peptides temporin A and indolicidin) was determined (Figure 2).

Both in the presence and in the absence of antimicrobial agents, the residual enzymatic activity of PVA-CG/His_6_-OPH (samples 2, 6, 10, and 14; Figure 2) was somewhat higher at zero time compared with the PVA-CG/BC/His_6_-OPH composite (samples 4, 8, 12, and 16; Figure 2). However, their activity became equal after 24 h in the presence of antimicrobial agents. The PVA-CG/BC/His_6_-OPH/indolicidin composite had the highest enzymatic activity among biomaterials with antimicrobial compounds (sample 16; Figure 2).

### 2.3. Antibacterial Activity of Composite Biomaterials 

**Figure 3:**
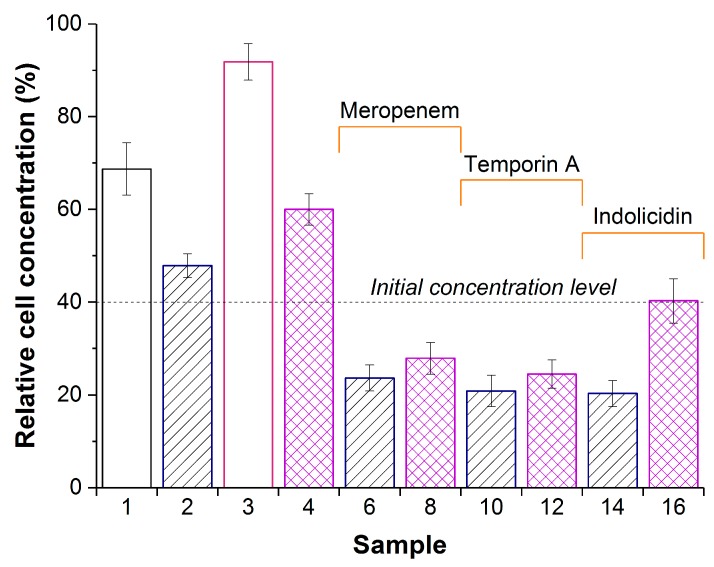
Concentration of *Pseudomonas* sp. in a suspension after 24 h exposure with PVA-CG (sample 1), PVA-CG/His_6_-OPH (samples 2,6,10, and 14), PVA-CG/BC (sample 3), or PVA-CG/BC/His_6_-OPH (samples 4,8,12, and 16) in the absence (samples 1,2,3, and 4) or in the presence of meropenem (samples 6 and 8), temporin A (samples 10 and 12), or indolicidin (samples 14 and 16). The dashed line indicates the cell concentration at zero time. Concentration of cells in the control without any additions after 24 h was assumed as 100%. Cultures treated by composite samples without His_6_-OPH but with antimicrobial agents for 24 h had almost the same bacterial concentration as the initial level.

The antibacterial activity of composite biomaterials was evaluated with *Pseudomonas* sp. as the G(-) cells (Figure 3).

The constant concentration of bacterial cells during exposure may be interpreted as some bacteriostatic effect, while the decrease in cell concentration is likely indicative of bactericide mode of action. From this standpoint, eight samples—namely, PVA-CG/His_6_-OPH (sample 2), PVA-CG/meropenem (sample 5), PVA-CG/BC/meropenem (sample 7), PVA-CG/temporin A (sample 9), PVA-CG/BC/temporin A (sample 11), PVA-CG/indolicidin (sample 13), PVA-CG/BC/indolicidin (sample 15), and PVA-CG/BC/His_6_-OPH/indolicidin (sample 16)—were certainly bacteriostatic. Five samples, namely—PVA-CG/His_6_-OPH/meropenem (sample 6), PVA-CG/BC/His_6_-OPH/meropenem (sample 8), PVA-CG/His_6_-OPH/temporin A (sample 10), PVA-CG/BC/His_6_-OPH/temporin A (sample 12), and PVA-CG/His_6_-OPH/indolicidin (sample 14)—had bactericide effect and were almost equal and undistinguishable under these experimental conditions.

## 3. Discussion

Samples of composite materials based on BC, PVA-CG, and His_6_-OPH in the presence and in the absence of various antimicrobial agents were prepared in this study, and their biocatalytic as well as antibacterial properties were evaluated.

The immobilization of His_6_-OPH on various BC samples showed that an increase in the porosity of BC fibers produced using fructose led to an increase in the activity of the immobilized enzyme. Obviously, widening the pores and/or increasing the surface area allowed the easiest enzyme penetration into the carrier. Similar results were obtained when the laccase was immobilized on BC of varying physicochemical and mechanical characteristics [27]. All of the BC types could be good carriers for laccase; however, the highest activity of immobilized enzyme was estimated for BCs with high porosity [27]. Most sorbents, including BC, can both adsorb and absorb biomolecules simultaneously, and it is almost impossible to distinguish between them [33]. Nevertheless, the strength of binding within the carrier matrix could be stronger as there is a physical barrier for absorbate elution. Such a barrier can be additionally created, and PVA-CG is one of the best polymers for that purpose [14].

Previously, the highest activity of immobilized His_6_-OPH was obtained in the case of wheat straw compared to other multiple cellulose-containing carriers [33]. The structure of the outer envelope of such a carrier is formed by long fibers of ca. 10–20 μm diameter, while the inner hollow is pierced by thin-walled channels up to 10–20 μm wide [34]. Synthetic PVA-CGs used in the work had the same internal structures [14,15]. Therefore, biomaterials based on PVA-CGs were expected to mimic the natural ones. The biggest differences between natural and (semi)synthetic carriers are the controllable procedures of obtaining them and variable characteristics. Moreover, strengthening these PVA-CGs by BCs with their own high sorption capacity would result in developing a promising carrier for His_6_-OPH immobilization, and this is quite intriguing.

These expectations were fulfilled, and more than 60% of His_6_-OPH was successfully entrapped in the PVA-CG and PVA-CG/BC composites. Interestingly, the introduction of BC into the macroporous PVA-CG matrix influenced the enzyme immobilization efficiency (Figure 2). However, at the same time, comparison of PVA-CG/BC/His_6_-OPH and BC/His_6_-OPH showed identical results, indicating that the presence of PVA-CG itself had little or no effect on the sorption immobilization of His_6_-OPH within BC. It seems that modification of the internal structure of PVA-CG macropores by BC (Figure 1f) is more significant than previously assumed. This could be an interesting topic for further research.

Based on the catalytic data alone, one can conclude that the PVA-CG/BC/His_6_-OPH/indolicidin composite is the best choice (Figure 2). Actually, the enzyme activity was already aligned between the different composites at 24 h, and based on antibacterial activity measurements, there was a strong negative influence of BC (Figure 3) in spite of the significantly improved antibacterial activity of His_6_-OPH/indolicidin [8]. Indolicidin has three positively charged amino acids (arginines and lysine) and can electrostatically interact not only with enzyme but also with BC. Unexpectedly, its bioavailability could be decreased in this way, and this is something that should be taken into account in future research.

Another interesting result of this work is that most samples that did not contain His_6_-OPH could not lead to the death of bacterial cells and only had a bacteriostatic effect (Figure 3), whereas samples showed a significant bactericidal effect in the presence of His_6_-OPH. This enzyme is able to inhibit the QS of bacterial populations and thus effectively improve the action of antimicrobial agents. The combination of multipurpose effectors in a single composite biomaterial could be a novel trend to fight complicated pathological conditions.

## 4. Materials and Methods

### 4.1. Materials

The following substances and preparations were used as received: PVA with a molecular weight of 86 kDa and a degree of deacylation of 100% (Acros Organics, Pittsburgh, PA, USA), indolicidin and temporin A (both from AnaSpec, Fremont, CA, USA), and N-(3-oxooctanoyl)-D,L-homoserine lactone (Sigma-Aldrich, Darmstadt, Germany). Deionized water was used to prepare all aqueous solutions.

Recombinant *Escherichia coli* SG13009[pREP4] cells were used to produce His_6_-OPH. The enzyme was isolated and purified using Ni-NTA agarose (Sigma-Aldrich, Darmstadt, Germany) as published elsewhere [35]. The purified His_6_-OPH was characterized as described earlier [36] in protein concentration determined by Bradford assay with Coomassie Brilliant Blue G-250 (Sigma-Aldrich, Darmstadt, Germany). The protein purity was confirmed by sodium dodecyl sulfate polyacrylamide gel (12%) electrophoresis using Mini-PROTEAN II cell (Bio-Rad, Hercules, CA, USA) followed by Coomassie Brilliant Blue R-250 (Sigma-Aldrich, Darmstadt, Germany) staining.

The enzymatic activity was measured spectrophotometrically at 405 nm with an Agilent 8453 UV–visible spectroscopy system (Agilent Technology, Waldbronn, Germany) equipped with a thermostated analytical cuvette and using 10 mM paraoxon (Sigma-Aldrich, Darmstadt, Germany) as a substrate. The reaction was realized in a 100 mM Na-carbonate buffer (pH 10.5) with His_6_-OPH concentration in a cuvette of ca. 7 × 10^−9^ M. One unit of enzyme activity (U) was defined as the quantity of the enzyme necessary to hydrolyze 1 μmol of paraoxon per min at 25 °C. The purity of His_6_-OPH preparation obtained (MW ≈ 37 kDa) was ca. 98%.

BC was produced and purified in accordance with the procedure described earlier [37]. To accumulate the bacterial biomass of *Komagataeibacter xylinum* B-12429 and for its immobilization in PVA-CG, Hestrin–Schramm (HS) nutrient medium (pH 6.5) with a variable main carbon source was used as follows (in g·L^−1^): carbon source, 20; yeast extract, 5; tryptone, 5; K_2_HPO_4_, 2.7; MgSO_4_, 0.5; citric acid, 1.15. The process was carried out at 28 ± 1 °C, 180 rpm for 19 ± 1 h. Harvested bacterial biomass was immobilized within PVA-CG using a patented method [38]; the cell concentration in the resultant immobilized biocatalyst was 30 wt %. Fructose, glycerol, Jerusalem artichoke hydrolyzate, or beet molasses were used as the main carbon sources. Jerusalem artichoke was hydrolyzed for 12 h at 50 °C and natural pH of the raw materials with constant mixing (200 rpm). Commercial enzymes—namely, inulinases of *Aspergillus niger* (Sigma-Aldrich, city, Germany), exoinulinases of *Penicillium verruculosum*, and β-glucosidase (Novozyme, city, Denmark)—were introduced in equal mass ratio into the processed raw material to a final concentration of 6 mg protein per 1 g of dry substrate. Next, the biosynthesis of BC in the media with different carbon sources was carried out under static conditions in flasks or a plastic container for 6 days at 28 °C. After that, BC fibers were washed from the culture broth with a 1 M KOH for 8 h and then with distilled water to neutral pH values. Next, they were dried at room temperature until a constant weight. The characteristics of the BC samples (Table 1) were evaluated according to previously described methods [37].

Complexes of His_6_-OPH with different antimicrobial agents were prepared as described previously [7] with minor modifications. In brief, antimicrobial peptides at a concentration of 0.13 g·L^−1^ (i.e., 68.3 μM indolicidin and 96.9 μM temporin A) or meropenem at a concentration of 1 g·L^−1^ (5.2 mM) in a 50 mM K-phosphate buffer (pH 7.5) containing 150 mM NaCl were mixed with 2 g·L^−1^ His_6_-OPH (28 μM) in the same buffer at 1:1 volume ratio and exposed for 30 min at room temperature.

### 4.2. Preparation of Immobilized Biocatalysts

#### 4.2.1. His_6_-OPH Immobilization via Absorption onto BC

To immobilize His_6_-OPH on different BC fibers, a diluted solution of purified enzyme with an activity of 300 U·mL^−1^ was used. BC samples were cut into 1 cm × 1 cm (4 ± 0.03 mg) pieces, and an enzyme solution (50 μL) was loaded on them (Figure 1) and exposed for 6 h at +8 °C. The weight of BC samples after His_6_-OPH application was 15 ± 1 mg (average of 5 measurements). Thus, the volume of enzyme solution absorbed by the BC samples was ~11 μL, which is equal to the immobilization of 825 U per 1 g of dry BC. After enzyme immobilization, BCs were washed for 10 min with a 100 mM Na-carbonate buffer (pH 10.5), and the activity of the immobilized enzymes was measured.

#### 4.2.2. His_6_-OPH Immobilization via Entrapment in the PVA-CG/BC Composite

BC was crushed in a mechanical mill to particles of ca. 2 mm × 2 mm and then suspended at 0.4 wt % in a 120 g·L^−1^ aqueous PVA solution. His_6_-OPH or its complex with antimicrobial agents was added to the resulting suspension under gentle stirring. The mixture was poured in portions of 0.9 g in plastic moulds with an inner diameter of 10 mm and a height of 5 mm. Cryotropic processing was performed by freezing at −20 °C for 12 h followed by defrosting of the samples at a heating rate of 0.03 °C/min with the microprocessor of an FP 45 HP programmed cryostat (Julabo, Seelbach, Germany). The prepared cryogels were exposed in a 100 mM Na-carbonate buffer (pH 10.5) for 24 h, and their residual enzymatic activity was determined.

### 4.3. Measurement of Enzyme Activity Toward AHLs

To determine the AHL-degrading activity of composites containing His_6_-OPH, a stock solution of 1 mM N-(3-oxooctanoyl)-D,L-homoserine lactone (C8-HSL) (Sigma-Aldrich, Darmstadt, Germany) in DMSO was incubated with samples at pH 7.5 and 25 °C for 6 h. The reactions were analyzed by a previously described method [7].

The data are presented as means of at least three independent experiments ± standard deviation (± SD). Statistical analysis was realized using SigmaPlot (version 12.5, Systat Software Inc., San Jose, CA, USA).

### 4.4. Measurement of Antibacterial Activity

Antimicrobial activities of composite biomaterials were investigated with *Pseudomonas* sp. 78G as the model G(-) bacteria. Cells were grown in a Luria–Bertani broth (10 g·L^−1^ tryptone, 5 g·L^−1^ yeast extract, 10 g·L^−1^ NaCl) at 28 °C using a shaking incubator with 180 rpm for 8 h. Suspension of *Pseudomonas* sp. at a concentration of 10^6^ cells·mL^−1^ was incubated with 100 g·L^−1^ composite biomaterials at 37 °C in a shaking incubator with 180 rpm for 24 h. Tubes containing bacterial cells or media without material samples were used as controls. To evaluate the residual concentration of viable cells, the optical density of the samples was determined at 560 nm using the Agilent 8453 UV–visible spectroscopy system (Agilent Technology, Waldbronn, Germany).

### 4.5. Characterization of PVA-CG/BC Composites

To investigate mechanical characteristics, the composite biomaterials were formed in dismountable cylindrical duralumin containers with a 15 mm internal diameter and a 10 mm height. Compression Young’s moduli (*E*_c_) were determined with the TA-Plus automatic texture analyzer (Lloyd Instruments Ltd., UK) from the linear region of the stress–strain curve at an uniaxial deformation rate of 0.3 mm·min^−1^. The measurements were performed up to a 30% deformation. Fusion temperature (*T*_f_) of the composites was measured as described earlier [20]. For this, a tightly sealed polyethylene test tube containing the material, with a metal ball located in the lower part of its column, was placed upside down into a water bath equipped with a stirrer. The temperature was elevated at a rate of 0.4 ± 0.1 °C·min^−1^. The temperature at which the ball passed through the layer of the fusing gel and fell onto the tube stopper was considered as the fusion temperature.

The elasticity modulus and fusion temperature of the composites were measured for three parallel samples, while the samples were prepared in three to five independent experiments. The obtained results were averaged.

To make SEM images, samples of BC, PVA-CG, and PVA-CG/BC/His_6_-OPH composites were freeze-dried with FreeZone 1 Liter Benchtop Freeze Dry System (Labconco, Kansas City, MO, USA), sectioned, sputtered by gold (by necessity), and studied with Supra 40-30-87 microscope (Carl Zeiss, Oberkochen, Germany) at various magnifications.

## 5. Conclusions

Various composite biomaterials based on PVA-CG, BC, and His_6_-OPH were produced in the presence of antimicrobial agents via a simple technique. Both PVA-CG and BC appeared to be important for the final characteristics of the biomaterials, with improved physicomechanical and thermal properties. From the catalytic and bactericidal efficiency standpoints, the most promising biomaterials seemed to be those combining His_6_-OPH with meropenem or temporin A. Such biomaterials can be interesting for biomedical applications as well as in other areas, such as covering, insulating, and making protective materials, including for biological usage.

## Figures and Tables

**Table 1:** Characteristics of BC samples synthesized by *K. xylinum* B-12429 cells in the medium with different main carbon sources.

Characteristic	Main Carbon Source			
	Glycerol	Fructose	Jerusalem Artichoke Hydrolyzate	Beet Molasses
Humidity (%)	98.0 ± 0.2	98.2 ± 0.2	97.3 ± 0.1	97.2 ± 0.1
Thickness (μm)	45 ± 3	40 ± 5	70 ± 2	75 ± 3
Tensile strength (MPa)	50 ± 10	45 ± 10	80 ± 15	85 ± 15
Porosity (%)	83 ± 2	85 ± 2	78 ± 2	75 ± 2
Crystallinity (%)	76 ± 1	77 ± 1	79 ± 1	77 ± 1

**Table 2:** Influence of BC content on compression modulus of elasticity (*E*_c_) and on fusion temperature (*T*_f_) of PVA-CG and PVA-CG/BC composites.

BC Content (wt %) *	*E*_c_ (kPa)	*T*_f_ (°C)
0	5.39 ± 0.24	72.1 ± 0.4
0.17	7.94 ± 1.7	73.2 ± 0.2
0.34	13.3 ± 1.4	73.5 ± 0.5
0.52	22.2 ± 2.6	74.7 ± 0.2
0.69	21.1 ± 1.0	76.3 ± 0.3
1.10	21.6 ± 0.6	84.0 ± 0.5

* Calculated by the dry matter amount.

**Table 3:** Activity of His_6_-OPH immobilized into different BC samples.

Carbon Source for BC	Specific Activity (U·g^−1^ of dry BC)	Residual Activity (%)
Fructose	536 ± 16	65 ± 2
Glycerol	487 ± 24	59 ± 3
Jerusalem artichoke hydrolyzate	437 ± 15	53 ± 2
Beet molasses	404 ± 8	49 ± 1
